# Assessing the efficacy and reproducibility of four common repetitive transcranial magnetic stimulation protocols: A sham-controled study

**DOI:** 10.1162/IMAG.a.1231

**Published:** 2026-07-16

**Authors:** Brice Passera, Peter J. Fried, Justine Magnuson, Stephanie S. Buss, Alvaro Pascual-Leone, Recep A. Ozdemir, Mouhsin M. Shafi

**Affiliations:** Berenson-Allen Center for Noninvasive Brain Stimulation, Department of Neurology, Beth Israel Deaconess Medical Center, Boston, MA, United States; Department of Neurology, Harvard Medical School, Boston, MA, United States; Department of Psychology, University of British Columbia Okanagan, Kelowna, BC, Canada; Deanna and Sidney Wolk Center for Memory Health and Hinda and Arthur Marcus Institute for Aging Research, Hebrew SeniorLife, Boston, MA, United States

**Keywords:** repetitive transcranial magnetic stimulation, electroencephalography, reproducibility, motor task, cortical excitability, TMS-evoked potential, plasticity

## Abstract

Repetitive transcranial magnetic stimulation (rTMS) has been widely used in both clinical and research settings to modulate brain activity and assess brain plasticity. The effects of rTMS are generally believed to involve modulation of cortical excitability; protocols such as 10 Hz and iTBS increase cortical excitability, while 1 Hz and cTBS are considered inhibitory. However, recent meta-analyses and studies have shown a wide array of interindividual responses to each protocol and poor reliability of the results. Here, we applied two sessions of the four most commonly used rTMS protocols (iTBS, cTBS, 10 Hz, 1 Hz) and sham to the primary motor cortex and assessed their neurophysiological and behavioral effects. We hypothesized that relative to sham, 10 Hz and iTBS would increase motor excitability and modulate motor response times, while 1 Hz and cTBS would have opposite effects. We recorded single-pulse TMS-EEG-EMG pre-rTMS and 5 and 25 minutes post-rTMS. Participants performed a sequential finger-tapping task (SFTT) 20 minutes pre-rTMS and 15 minutes post-rTMS. Our main outcome measures were P30 amplitude, early LMFP (15–75 ms) area under-the-curve, MEP, and SFTT completion time. We calculated the Intra-Class Correlation between each session for each measure. For each protocol, we found no consistent protocol-dependent group effect on MEPs, TEPs, or behavior, with high intra-individual variability across sessions. An increase in SFTT completion time occurred for all active protocols but also sham. The test–retest reliability of rTMS effects was low (ICC < 0.5) for almost all protocols and metrics. This study is the first comprehensive assessment of the effects and reliability of the most commonly used rTMS protocols on cortical excitability and behavior. Our results suggest that rTMS effects are not reliable, at least at the single-session level, and that the inclusion of sham rTMS is essential for identifying protocol-specific effects. These results also suggest that a fundamental and unbiased re-evaluation of potential rTMS mechanisms is necessary.

## 1 Introduction

Repetitive transcranial magnetic stimulation (rTMS) is increasingly utilized to obtain insights into brain function and as a therapeutic approach for a broad array of neuropsychiatric disorders. Conventional models posit that rTMS affects brain function through modulation of local cortical excitability, leading to changes in cortical network activity or connectivity (Klomjai et al., 2015; Ziemann et al., 2001). Early studies suggested that high-frequency (≥5 Hz) rTMS increased cortical excitability and could improve performance on tasks involving the stimulated region, whereas low-frequency (≤1 Hz) rTMS decreased cortical excitability and might impair performance (Chen et al., 1997; Lefaucheur et al., 2014; Pascual-Leone et al., 1994) (although paradoxical functional facilitation and baseline cortical excitability could also impact the behavioral or cognitive effects of rTMS) (Silvanto et al., 2008; Théoret et al., 2003). More recently, shorter theta-burst TMS protocols, involving pulses delivered in gamma-frequency bursts (e.g. 50 Hz) administered at theta frequencies (e.g. 5 Hz), were developed (Huang et al., 2005; Larson & Lynch, 1989); initial studies reported that intermittent theta-burst stimulation (iTBS) increased cortical excitability, whereas continuous theta-burst stimulation (cTBS) decreased excitability.

However, it has recently become apparent that the mechanisms of rTMS effects, and their reliability, may not be definitively established. Specifically, a growing body of studies on the TBS protocol failed to identify the expected modulation in MEPs (Boucher et al., 2021; Maeda et al., 2000; Magnuson et al., 2023), and, more recently, also failed to identify any reliable single-session modulation of TMS-evoked EEG potentials (TEPs) (Carrette et al., 2024; Ozdemir et al., 2021). Furthermore, studies assessing the test–retest reproducibility of TBS effects also showed high inter- and intra-individual variability (Nettekoven et al., 2015; Schilberg et al., 2017). One resulting unanswered question is whether poor reliability is restricted to TBS, or whether conventional low- (e.g. 1 Hz) and high-frequency (e.g. 10 Hz) rTMS protocols also lack reliable effects (Beynel et al., 2019; Fried et al., 2017; Maeda et al., 2000; Ozdemir et al., 2021; Prei et al., 2023). Furthermore, very few studies have investigated the reliability of rTMS effects on task performance (Beynel et al., 2019; Chou et al., 2020). Thus, it is unclear whether modulation of cortical excitability is the fundamental physiologic mechanism of rTMS and is responsible for its behavioral effects. As a mechanistic understanding of rTMS effects on brain and behavior could lead to the development of more effective protocols, addressing this knowledge gap could have a substantial impact on this developing field.

In this study, we comprehensively assessed the test–retest reproducibility of rTMS protocols on cortical excitability, corticospinal excitability, and motor processes. We tested four active protocols—two most common clinical frequencies (1 Hz, 10 Hz) and two theta-burst protocols (iTBS, cTBS)—along with a control protocol (SHAM), measuring MEPs, TEPs, and motor performance on a Sequential Finger Tapping Task (SFTT). Based on the literature (Kobayashi et al., 2004, 2009), we hypothesized that TEPs would be suppressed by 1 Hz and cTBS and facilitated by 10 Hz and iTBS compared with SHAM. Additionally, we expected that 1 Hz and cTBS would decrease the ipsilateral average completion time of a finger-tapping sequence, while 10 Hz and iTBS would increase it. Lastly, we hypothesized that neurophysiological and motor rTMS effects would be correlated, but anticipated these effects would not be reproducible across sessions.

## 2 Material and Methods

### 2.1 Participants

Twenty-eight participants (10 females, mean age 39 ± 16 years) completed the study. All met the inclusion criteria: no history of neurological diseases, major systemic illnesses, major psychiatric conditions, or substance abuse, as assessed by the Mini-International Neuropsychiatric Interview (MINI); and no contraindications to TMS or MRI (Rossi et al., 2021). Written informed consent was obtained from all participants, and the study was approved by the institutional review board of Beth Israel Deaconess Medical Center (Boston, MA).

### 2.2 Study design

Before the TMS session, participants had two screening visits where they consented, underwent baseline neuropsychological assessment and a T1-MRI scan, and completed training for the SFTT. The next 10 sessions were divided into 2 blocks of 5 sessions for the 5 different protocols: 1 Hz, 10 Hz, iTBS, cTBS, and SHAM. The order of the different rTMS protocols was randomized across participants in the first block, with each participant repeating their assigned protocol sequence in the second block. There was a minimum 1-week gap between each session, with at least a month-long interval between blocks. Realistic sham rTMS was delivered using the A/P Cool B65 coil (MagVenture) positioned in placebo mode with peripheral stimulation. The sham protocol randomly mimicked the rTMS parameters of one of the four active protocols for each participant. Sessions were scheduled consistently at the same time of day and, for female participants, aligned with their menstrual cycle.

**Fig. 1.**
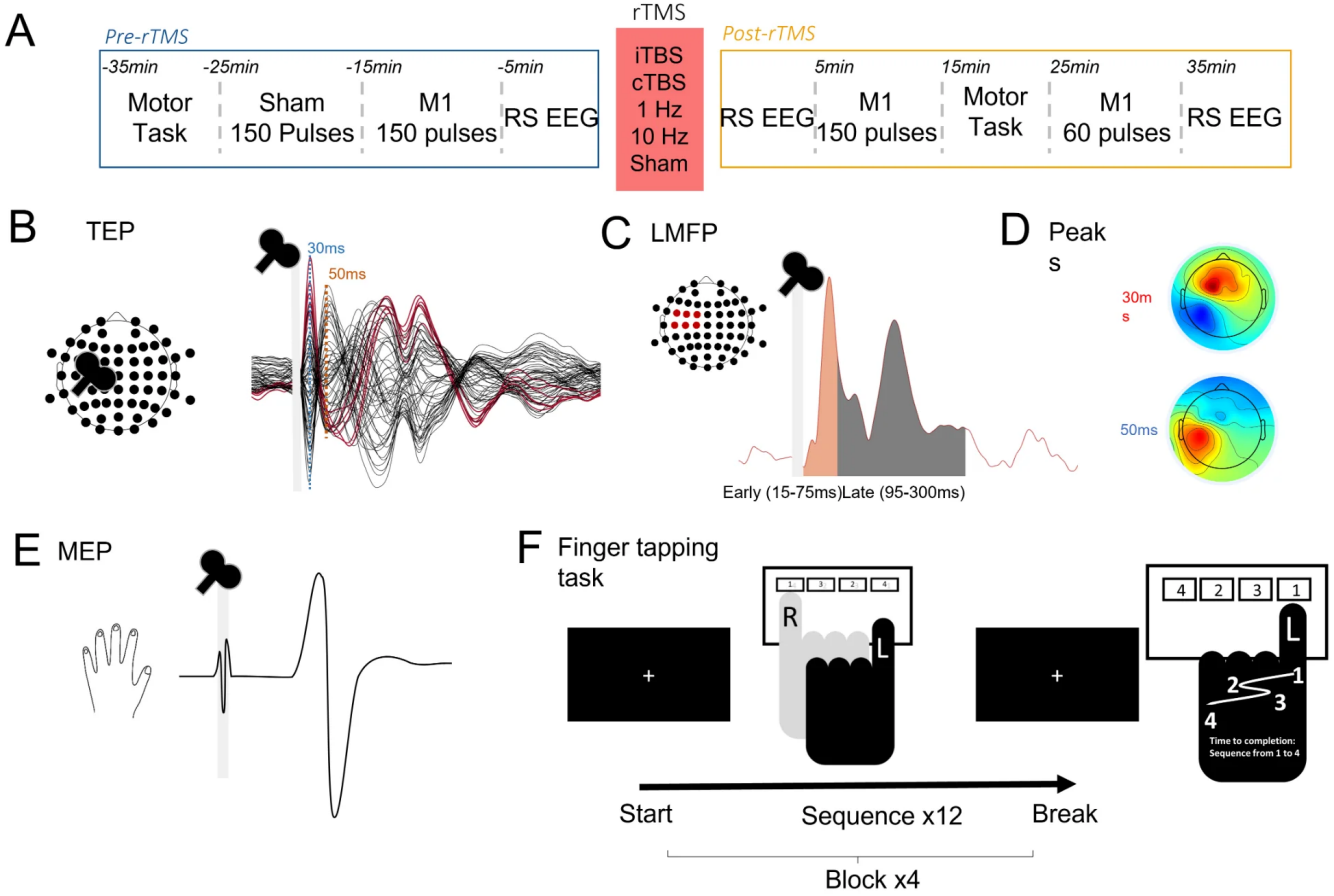
(A) Study design—Organization of an experimental session. (B) Butterfly plot of a TEP with relevant peaks. (C) Detail of LMFP analysis, with electrode locations for the region of interest (in red). Example of an LMFP with the time of interest for “Early” LMFP in beige and for “Late” LMFP in gray. (D) Example of topographies for relevant peaks (P30 and N45) of a TEP. (E) Example of MEP. (F) Schematic summary of the SFTT, organization of the experiment (4 blocks of 12 sequences) and the finger tapping sequence.

Each session followed the same structure (Fig. 1A). We first recorded baseline resting-state EEG in two blocks of 5 minutes (eyes open and eyes closed). Then the participant performed the training SFTT (Fig. 1F); training and subsequent testing were performed with each hand to separately characterize effects ipsilateral and contralateral to stimulation. Subsequently, neuronavigated TMS stimulation was initiated. First, the motor hotspot targeting the First Dorsal Interosseous (FDI) was identified, coil orientation was then adjusted to maximize MEPs amplitude and stability, followed by resting motor threshold (rMT) assessment; rMT was defined as the lowest stimulation intensity eliciting an MEP >50 µV in at least 5 out of 10 trials. For active motor threshold (aMT), participants were instructed to press their index finger against their thumb and maintain a stable contraction at ~10% of maximum effort and was defined as the lowest stimulation intensity eliciting MEPs of at least 200 µV and a cortical silent period in a minimum of 5 of 10 trials.

Subsequently, experimental data collection was started. The experimental blocks (Fig. 1A) were as follows: (1) resting-state EEG recordings, (2) baseline SFTT, (3) SHAM single pulse, (4) baseline single pulse TMS/EEG (150 single TMS pulses at 120% rMT), (5) rTMS protocol over the FDI hotspot, (6) T05 TMS/EEG (150 pulses), (7) T15 SFTT test, (8) T25 Single pulse TMS (60 pulses), (9) T35 resting-state EEG. Each rTMS protocol was parametrized based on prior studies (Turi et al., 2021) evaluating the efficiency of rTMS protocols based on stimulation intensity and number of pulses. TBS protocols were delivered as 3 pulse bursts at 50 Hz with 200 ms between bursts (for a total of 600 pulses) at 80% AMT. iTBS was delivered with a 2-second on and 8-second off pattern, while cTBS was delivered continuously for 40 seconds. Then, 10 Hz rTMS was applied at 120% RMT in 4-second trains, with a 26-second inter-train interval for 37.5 minutes (3000 pulses total). The 1 Hz rTMS was applied at 110% RMT as a continuous train for 15 minutes (900 pulses total).

### 2.3 TMS stimulation

TMS pulses were delivered using a figure-of-8 coil (MagPro Cool B-65) with a MagPro X100 stimulator (MagVenture A/S). Coil positioning was tracked using neuro-navigation (BrainSight, Rogue Research Inc.), with individual T1-weighted anatomical MRI (GE Healthcare, Ltd., United Kingdom) acquired prior to the first experimental session. EEG was recorded with 64 active electrodes cap and BrainVision ActiChamp (Brain Products, Gmbh). EMG for the FDI was recorded using the aux port of the ActiChamp with Ag-AgCl electrodes placed on a Tendon-belly montage. EEG and EMG data were recorded at 5 kHz and impedances were kept below 5 kΩ. Realistic sham was delivered using the A/P cool B65 (MagVenture) with active electrical stimulation through electrodes placed on the forehead between Fp1 and AF7; participants were asked to judge the electrical stimulation intensity to match as closely as possible the intensity of sensation produced by the TMS pulse. A 3D printed spacer was attached to the placebo side to minimize the E-field on the cortex (Opitz et al., 2014).

### 2.4 Sequential finger-tapping task

Participants performed the SFTT (E-prime) with each hand (Fig. 1F). The task consisted of using the index finger to press a sequence of four buttons (Ipsilateral Hand: 1,3,2,4; Contralateral Hand: 4,2,3,1) as quickly and accurately as possible. The experiment consisted of 4 blocks of 12 sequences. To reduce learning effects, the participants trained during the baseline/screening visit and had a dedicated retraining of eight blocks before the start of each TMS/EEG session. The main outcome measure for the SFTT was the completion time (CT) for each sequence averaged within the 12-sequence blocks.

### 2.5 EEG preprocessing

TMS-EEG data pre-processing was performed on Matlab (MathWorks Inc., USA) using EEGLAB (Delorme & Makeig, 2004), and customized scripts utilizing TESA functions through a semi-automatized process (Mutanen et al., 2020; Rogasch et al., 2014). The initial preprocessing was done with a custom fully automated pipeline (see below), and the resulting data were reviewed manually. If this initial processed data were not satisfactory, the data were reprocessed with manual pipeline. Following manual intervention, when the data quality remained unsatisfactory, the dataset was rejected. We only included in the final analysis participants with clean Baseline and T05 data for both visits 1 and 2, resulting in 1 Hz, 20 participants; 10 Hz, 21 participants; iTBS, 19 participants; cTBS, 20 participants; SHAM, 20 participants. We also processed the T25 TMS-EEG data; however, due to the low trials counts (60 pulses administered), we only included datasets with >40 trials after preprocessing, resulting in smaller sample sizes for T25 (1 Hz, 14 participants; 10 Hz, 11 participants; iTBS, 17 participants; cTBS, 15 participants; SHAM, 14 participants).

The automated manual pipeline followed these steps: data were epoched between -1 second and 2 seconds around the TMS pulse. Baseline correction was done by subtracting the mean pre-stimulus signal amplitude (-900 to -100) from the rest of the epoch in each channel and was followed by channel rejection. Data were then zero padded between -2 ms and 14 ms. All zero-padded epochs were then tagged based on voltage (>100 mV), kurtosis (>3), and joint probability (single channel-based threshold >3.5 sd; all channel-based threshold >5 sd) metrics to identify excessively noisy epochs and supplemented with visual inspection. Next a first round of ICA was executed to identify and remove early TMS artifacts. The previously zero-padded EEG data were then interpolated using linear interpolation, bandpass filtered using a forward–backward 4th order butterworth filter from 1 to 100 Hz, notch filtered between 57 and 63 Hz, and referenced to global average, then sub-epoched from 500 ms pre- to 1000 ms post-TMS. In the automated pipeline, an additional round of ICA was performed to automatically remove eye blinks. Then another round of ICA was performed to remove all remaining artifact components including remaining eye movement, muscle noise (EMG), single electrode noise, TMS-evoked muscle, and EKG (Ozdemir et al., 2021).

### 2.6 TMS-EEG metrics

Global Mean Field Power (GMFP) was computed from -200 ms pre-TMS to 400 ms post-TMS using the following equation:

$$GMFP\left(t\right)=\sqrt{\left\{\frac{\sum_{i}^{k}\left(\text{V}i\;\left(\text{t}\right)-{\text{V}}_{mean\left(t\right)}\right)2}{K}\right\}}$$

where Vi(t) is the voltage at electrode i at a certain point in time, V_mean(t)_ is the mean of instantaneous TEP across electrodes, and K is the number of electrodes. Local Mean Field Power (LMFP) was calculated as the root-mean-square value of the activity in the stimulated motor region of interest (electrodes (‘C1’,’C3’,’FC3’, ‘FC1’, ‘C5’, ‘FC5’)). For both LMFP and GMFP, we calculated the area under the curve for early (15–75 ms) time windows. The P30 peak was defined as the first positive peak in the time window from 25 to 45 ms following the TMS pulse. The primary outcome measures used for the analyses were P30 amplitudes and the amplitude of the early LMFP (Fig. 1C, D), corresponding to local brain activations in response to the TMS pulse. For supplemental analysis, N15-P30 was extracted using the first negative peak from 16 ms to 25 ms and the P30 from the P30 analysis. Additionally, to isolate local activity, we computed the LMFP and P30 using a Hjorth-C3 montage. Lastly, to control for the length of the post-rTMS stimulations block, we evaluated the amplitude of all the metrics above (AUC-LMFP, P30, N15-P30 amplitude) for the first 40 and last 40 trials of T5 separately, and compared each of them with the last 40 trials of baseline. All of these supplementary analyses are given in Supplementary Materials.

### 2.7 MEP processing

Details for the MEP processing are given in Magnuson et al. (2023). For this analysis, we restricted our analysis to only those participants with complete TMS-EEG data (see above).

### 2.8 Statistical analysis

Statistical analysis was done using general linear models (The Jamovi Project, 2024) with subject as random intercept. We analyzed rTMS effects for each measure (P30, early LMFP, and MEP), including Visit (1,2) and time (baseline, post-rTMS) as independent variables. We computed the main effect for each Visit and Time as well as the interaction between Time and Visit. For behavior, that is., contralateral/ipsilateral CT, to compare against non-active intervention, we included protocol as an independent variable (active vs sham) and thus included the interaction Protocol x Time in the model. Non-normal data (Shapiro–Wilk test) were log transformed. We calculated pre/post rTMS ratios (T05/Baseline) for each protocol to evaluate rTMS efficacy, assess baseline excitability effects, and correlate measures using Spearman’s Rho. The same analysis was conducted with T25 data as a secondary investigation. Each protocol was first analyzed independently due to the imbalance in sample size between protocols. We also ran supplemental omnibus general linear models including all protocols in the models as a main variable along with all the other variables from the protocol-specific models on all our outcome variables for TEPs, MEPs, and behavior. These models were also run on the second supplementary analyses including as a “Condition” variable that blocks of trials were used to compute the TEP (last 40 of baseline, first 40 of T5, last 40 of T5) along with the other variables. The supplemental analyses results are given in Supplementary Materials.

Test–retest reliability was assessed via intraclass correlations between Visits 1 and 2 ratio scores. Following convention (Koo & Li, 2016), ICCs were classified as <0.25 “very low,” 0.25–0.50 “low,” 0.50–0.75 “moderate,” and >0.75 “high” reproducibility.

For comparison with the literature, we categorized participant responses as “Facilitation” (≥10% increase), “Suppression” (≥10% decrease), or “No Change” (within ±10% of baseline (Nettekoven et al., 2015)). The influence of baseline excitability on rTMS effects was examined using Spearman correlations between pre-TMS measures and ratio scores, with reproducibility assessed via Fisher Z comparisons. Bonferroni correction addressed multiple comparisons.

In an exploratory data-driven analysis of rTMS effects on TEPs, we used cluster–permutation techniques on paired sample t-test statistics to compare GMFP and LMFP at each time point pre- versus post-rTMS (baseline vs T05, vs T25). These analyses were also run to compare the 40-trial TEPs to verify the evolution of TEPs within the T5 block across the full time series. Each visit and protocol were analyzed separately. Paired sample t-tests were computed for each time point to identify significant differences between baseline and T05 TEPs, and the t-values from consecutive significant time points were summed to determine the cluster sum. Permutation t-tests (n = 1000) were conducted by randomly shuffling 50% of subjects across conditions (baseline vs T05, vs T25), and real clusters were defined as significant if their size and cluster sum exceeded 95% of the value obtained from the permuted samples.

## 3 Results

### 3.1 TMS-EEG results

**Fig. 2.**
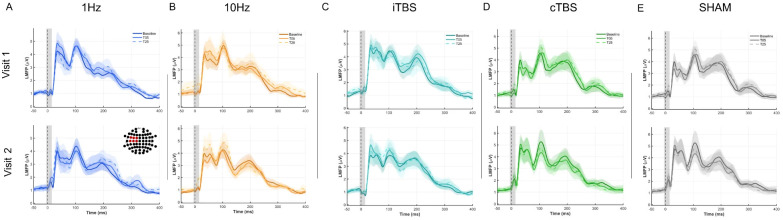
Group average LMFP for Visit 1 (Top row) and Visit 2 (Bottom row) for each protocol (1 Hz – A; 10 Hz – B; iTBS – C; cTBS – D; SHAM – E). Each plot shows both the baseline and T05 LMFPs with T25 represented as a dotted line due to lower sample size with the shading representing standard deviation.

LMFPs before and after each rTMS intervention are shown in Figure 2. Based on the literature, our primary hypothesis was that rTMS would modulate the early components of the TEP (Ozdemir et al., 2021); thus, our main analyses focused on the P30 amplitude in the peaks analysis, and the area under the curve of the early potentials (15 to 75 ms) for the LMFP.

#### 3.1.1 P30 analysis

**Fig. 3.**
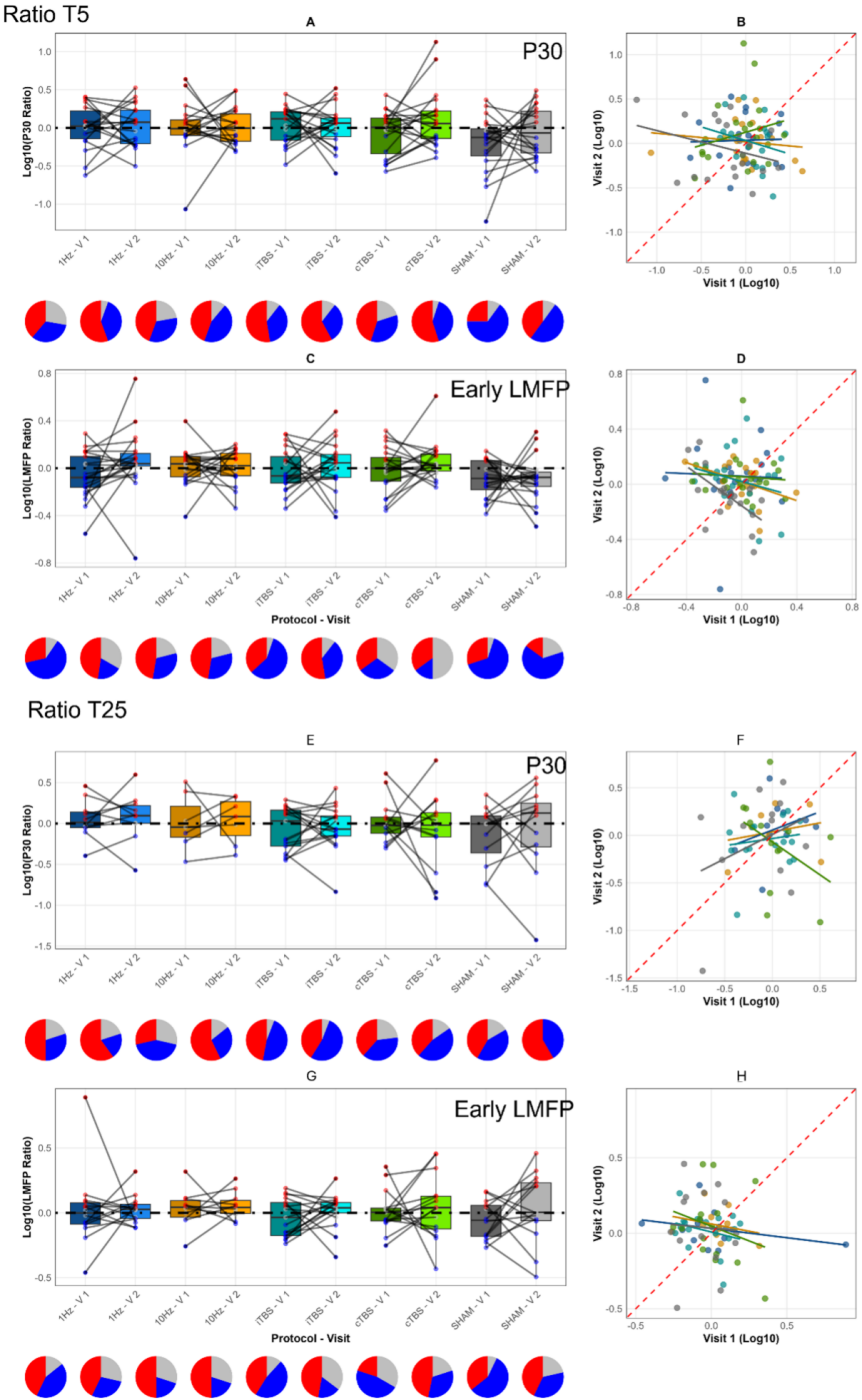
Effect of rTMS on TMS/EEG measures. (A) Plot of the log_10_ of the ratio between pre- and post-rTMS for both visits (V1 - darker, V2 - lighter shade) and each protocol (1 Hz, blue - 10 Hz, orange - SHAM, gray). The horizontal dotted line represents a ratio of 1, that is, no change. For each condition, data for each participant are plotted and linked between V1 and V2 with a line. Blue dots indicate inhibition of at least 10% compared with baseline, red dots indicate an increase of at least 10%, and gray dots indicate no change. The distribution of responses with inhibition, facilitation, and no change is depicted in the pie chart below each boxplot. (B) Results for early LMFP. (C) Scatter plot of P30 ratios (log_10_) of the ratio between baseline and T05 for V1 (x-axis) and V2 (y-axis) for 1 Hz (blue), 10 Hz (yellow) and SHAM (black). (D) Scatter plot for early LMFP ratios (log_10_). (E) Plot of the log_10_ of the ratio between pre- and T25 rTMS for both visit and each protocol (F) Results for early LMFP. (G) Scatter plot of P30 ratios (log_10_) of the ratio between baseline and T25 for V1 (x-axis) and V2 (y-axis). (H) Scatter plot for early LMFP baseline and T25 ratios (log_10_).

In the P30 analysis at T05 (Fig. 3), there was no significant difference in amplitude between baseline and post-rTMS (time) for conventional protocols (10 Hz [F(1,54) = 0.834, p = 0.365], 1 Hz[F(1,57) = 0.035, p = 0.852]), TBS protocols (iTBS [F(1,53) = 0.280, p = 0.599], cTBS [F(1,56) = 0.141, p = 0.708]), and SHAM [F(1,56) = 0.489, p = 0.487]. Post hoc analysis for Hjorth-C3 montage also showed no significant effects of time for either protocols (see Supplementary Materials).

Visit effects varied across protocol groups: conventional protocols showed no difference between visits (10 Hz [F(1,56) = 1.215, p = 0.275], 1 Hz[F(1,57) = 1.34, p = 0.251]), while TBS protocols showed mixed results with iTBS demonstrating a significant effect of Visit [F(1,53) = 16.46, p < 0.001, where Visit 2 showed an increase of 0.197 μV compared with Visit 1)] but cTBS showing no effect [F(1,56) = 1.56, p = 0.216]. SHAM also showed no visit effect [F(1,56) = 1.217, p = 0.275].

No interactions were observed for conventional protocols (10 Hz [F(1,55) = 0.883, p = 0.351], 1 Hz[F(1,56) = 0.01, p = 0.908]), TBS protocols (iTBS [F(1,54) = 0.00, p = 0.994], cTBS [F(1,56) = 0.51, p = 0.474]), and SHAM [F(1,56) = 2.15, p = 0.148].

Test–retest reliability of individual rTMS effects from Visit 1 to Visit 2 was poor across all protocol groups (Fig. 3, Table 1; 1 Hz ICC = -0.13, 10 Hz ICC = -0.12, SHAM ICC = -0.29, iTBS ICC = -0.27, cTBS ICC = 0.22). Post hoc exploration of the direction of effect (i.e., facilitation or suppression, Fig. 3) showed that individual participants often responded inconsistently across sessions for all protocols, with similar distributions of effects between visits but poor reproducibility at the individual level.

**Table 1.** Intra-class correlation between Sessions 1 and 2 for each protocol and outcome measures.

Protocol	P30	LMFP	Contralateral	Ipsilateral	MEP	P30_T25*	LMFP_T25*
1 Hz	-0.13	-0.05	0.27	-0.30	0.20	-0.04	-0.13
10 Hz	-0.12	-0.39	0.07	0.52	0.27	0.06	-0.25
SHAM	-0.29	-0.33	0.28	0.04	0.30	0.34	-0.03
iTBS	-0.27	-0.23	0.05	0.28	-0.39	0.14	-0.28
cTBS	0.22	-0.08	0.14	0.29	-0.4	-0.49	-0.26

Line represents different protocols while columns represent each of the outcome measure.

* Smaller sample size.

#### 3.1.2 Early LMFP

There was no significant time effect on the early LMFP at T05 for conventional protocols (Fig. 3.: 10 Hz [F(1,54) = 0.354, p = 0.554], 1 Hz [F(1,60) = 0.004, p = 0.948]), TBS protocol iTBS [F(1,54) = 0.052, p = 0.819], and SHAM [F(1,57) = 1.279, p = 0.263] (Fig. 3). However, TBS protocol cTBS showed a significant main effect of time with an increase in the LMFP after stimulation [F(1,52) = 32.72, p < 0.001], where post cTBS showed a significantly higher AUC-LMFP than baseline (+0.584, SE = 0.104).

Visit effects were protocol specific: conventional protocols showed no differences between visits (10 Hz [F(1,54) = 0.376, p = 0.542], 1 Hz [F(1,60) = 1.365, p = 0.247]), while TBS protocols both demonstrated significant visit effects (iTBS [F(1,54) = 10.56, p < 0.01, where Visit 2 was significantly higher than Visit 1], cTBS [F(1,52) = 6.81, p = 0.012, Visit 2 was significantly higher than Visit 1]), and SHAM showed no visit effect [F(1,57) = 0.611, p = 0.438].

Interactions were found only for TBS protocol cTBS [F(1,52) = 31.47, p < 0.001], where post hoc analyses of the effect reveal the effect predominantly at V2 baseline vs post cTBS (+0.859, SE = 0.07), while conventional protocols (10 Hz, 1 Hz), iTBS [F(1,54) = 0.29, p = 0.590], and SHAM showed no interactions. Post hoc analysis for cTBS revealed no significant difference between pre- and post-stimulation in Visit 1 [F(1,52) = 0.0796, p_bonferroni = 1.00], but a significant increase in the LMFP after cTBS in Visit 2 [F(1,52) = 7.87, p_bonferroni < 0.001].

Despite these statistical effects, ICC computations showed poor test–retest reliability across all protocol groups (Fig. 3; Table 1; 1 Hz ICC = -0.05, 10 Hz ICC = -0.39, SHAM ICC = -0.33, iTBS ICC = -0.23, cTBS ICC = -0.08), and participants had inconsistent response patterns across sessions regardless of protocol type. Post hoc analysis for Hjorth-C3 montage showed no significant effects (see Supplementary Materials and Supplementary Fig. S1).

#### 3.1.3 T25 analysis P30

In the P30 analysis at T25 (Fig. 3), there was no significant difference in amplitude between baseline and post-rTMS (Time) for conventional protocols (10 Hz [F(1,40) = 0.38, p = 0.541], 1 Hz [F(1,46) = 0.15, p = 0.696]), TBS protocols (iTBS [F(1,51) = 0.19, p = 0.665], cTBS [F(1,48) = 0.01, p = 0.914]), and SHAM [F(1,51) = 0.59, p = 0.443].

Visit effects differed across protocol groups: conventional protocols showed no significant differences between visits (10 Hz [F(1,40) = 0.05, p = 0.821], 1 Hz [F(1,46) = 2.44, p = 0.125]), while TBS protocols showed mixed results with iTBS demonstrating a significant difference between visits [F(1,51) = 19.5, p < 0.001, V2 > V1 +0.73, SE = 0.43] but cTBS showing no effect [F(1,46) = 1.84, p = 0.181]. SHAM also showed no visit effect [F(1,50) = 0.04, p = 0.839].

No interactions were observed for any protocol group: conventional protocols (10 Hz [F(1,39) = 0.01, p = 0.912], 1 Hz [F(1,45) = 0.05, p = 0.808]), TBS protocols (iTBS [F(1,51) = 0.136, p = 0.714], cTBS [F(1,47) = 0.113, p = 0.738]), and SHAM [F(1,45) = 0.02, p = 0.864].

ICC computation showed very low reproducibility for 1 Hz (ICC = -0.04), while conventional 10 Hz (ICC = 0.06), TBS protocols (iTBS ICC = 0.14, cTBS ICC = -0.49), and SHAM (ICC = 0.34) showed varied test–retest reliability (Fig. 3; Table 1). Post hoc exploration of the direction of effect showed that individual participants often responded inconsistently across sessions for most protocols.

#### 3.1.4 T25 analysis early LMFP

There was no significant time effect on the early LMFP at T25 for conventional protocols (10 Hz [F(1,42) = 1.19, p = 0.279], 1 Hz [F(1,53) = 0.0, p = 0.936]), TBS protocols (iTBS [F(1,53) = 0.04, p = 0.843], cTBS [F(1,47) = 0.163, p = 0.688]), and SHAM [F(1,49) = 0.35, p = 0.554] (Fig. 3).

Visit effects varied by protocol group: conventional protocols showed no differences between visits (10 Hz [F(1,42) = 0.33, p = 0.566], 1 Hz [F(1,49) = 0.04, p = 0.841]), TBS protocols showed protocol-specific effects with iTBS demonstrating a significant visit effect [F(1,53) = 7.53, p = 0.008, with V2 > V1 +62.72, SE = 22.8] while cTBS showed no effect [F(1,47) = 1.130, p = 0.293], and SHAM showed no visit effect [F(1,45) = 1.45, p = 0.234].

No interactions were found for any protocol group: conventional protocols (10 Hz [F(1,42) = 0.00, p = 0.955], 1 Hz [F(1,49) = 0.03, p = 0.849]), TBS protocols (iTBS [F(1,53) = 0.36, p = 0.850], cTBS [F(1,47) = 0.333, p = 0.566]), and SHAM [F(1,45) = 0.08, p = 0.779].

ICC computations showed very low to low test–retest reliability across protocol groups: conventional protocols (1 Hz ICC = -0.13, 10 Hz ICC = -0.25), TBS protocols (iTBS ICC = -0.28, cTBS ICC = -0.26), and SHAM (ICC = -0.03) (Fig. 3; Table 1), and participants had inconsistent response patterns across sessions for most protocols.

#### 3.1.5 Unified mixed models

Additional analyses using a unified mixed-effects model including all five protocols simultaneously also demonstrated no significant effects of protocol, time, or visit, and critically no significant Protocol × Time interaction (see Supplementary Materials for detailed results). These findings corroborate our protocol-specific analyses and support our conclusion that the absence of reliable rTMS effects is consistent across all tested protocols.

#### 3.1.6 LMFP and GMFP data-driven analysis

For the data-driven exploratory analysis, we analyzed each protocol separately as the number of participants with complete data was not consistent between protocols. Cluster-based permutation analysis did not reveal any reliable modulation of either the LMFP (Fig. 2) or GMFP for any protocol or time point. We also examined TEPs using a C3-Hjorth montage to isolate activity under the site of stimulation and minimize volume conduction effects. Consistent with our primary ROI analysis, the C3-Hjorth montage revealed no significant effects of Time or Time × Visit for any protocol (all p > 0.05; see Supplementary Materials).

Supplementary analysis. The 40-trial block analysis revealed no evidence of differential effects in early versus late trials compared with baseline for any protocol or outcome measure (LMFP, P30, Hjort-C3) (see Supplementary Materials and Supplementary Figs. S2, S3, S4 and for representative iTBS data see Supplementary Fig. S5).

### 3.2 Behavior

**Fig. 4.**
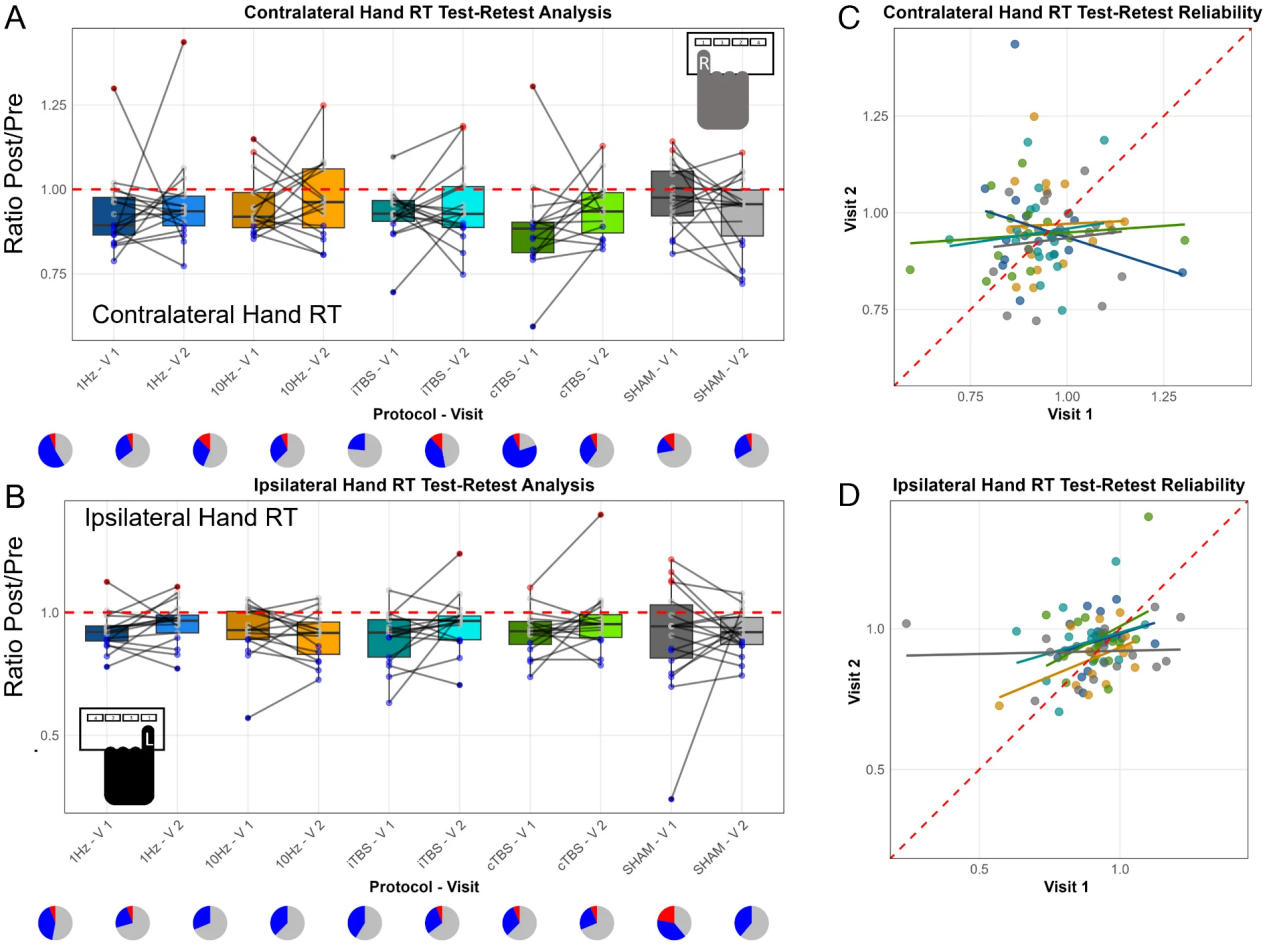
Effect of rTMS on SFTT. (A) Plot of the log_10_ of the ratio between pre- and post-completion time for the contralateral hand rTMS for both visit (V1 - darker, V2 - lighter shade) and each protocol (1 Hz, blue - 10 Hz, orange - SHAM, gray). Horizontal dotted line represents a ratio of 1, that is, no change. For each condition, data for each participant are plotted and link between V1 and V2. Dots color represented in blue: a inhibition of at least 10% compared with baseline, in red and increase of at least 10% and in gray no change. The distribution of inhibition, facilitation, and no change is reported in the pie chart below each boxplot. (B) Ratio between pre- and post-completion time for the ipsilateral hand. (C) Scatter plot of RT contralateral hand ratios (log_10_) of the ratio between baseline and T05 for V1 (x-axis) and V2 (y-axis) for 1 Hz (blue), 10 Hz (orange), and SHAM (gray). (D) Scatter plot of RT ipsilateral hand (log_10_).

We evaluated the effects of rTMS on the SFTT (Fig. 4). Across all protocol groups, consistent main effects were observed: significant effects of visit (conventional protocols: 1 Hz F(1,371) = 56.65, p < 0.001, -21.70 ms V2 vs V1, 10 Hz F(1,375) = 34.38, p < 0.001, -18.49 ms V2 vs V1; TBS protocols: iTBS F(1,380) = 22.73, p < 0.001, -19.31 ms V2 vs V1, cTBS F(1,374) = 13.219, p < 0.001, -10.52 ms V2 vs V1), time (conventional protocols: 1 Hz F(1,370) = 30.06, p < 0.001, +16.82 ms post vs pre, 10 Hz F(1,375) = 38.76, p < 0.001, +19.07 ms post vs pre; TBS protocols: iTBS F(1,379) = 35.01, p < 0.001, +19.64 ms post vs pre, cTBS F(1,373) = 37.14, p < 0.001, +21.14 ms post vs pre), and hand (conventional protocols: 1 Hz F(1,370) = 28.165, p < 0.001, -17.05 ms ipsilateral vs contralateral, 10 Hz F(1,375) = 41.77, p < 0.001, -19.54 ms ipsilateral vs contralateral; TBS protocols: iTBS F(1,380) = 43.75, p < 0.001, -24.08 ms ipsilateral vs contralateral, cTBS F(1,373) = 35.23, p < 0.001, -21.05 ms ipsilateral vs contralateral).

Crucially, no main effect of protocol was found for any stimulation type (conventional protocols: 1 Hz F(1,370) = 0.14, p = 0.705, -4.32 ms vs SHAM, 10 Hz F(1,375) = 1.44, p = 0.230, -6.14 ms vs SHAM; TBS protocols: iTBS F(1,370) = 0.03, p = 0.855, +0.16 ms vs SHAM, cTBS F(1,375) = 0.32, p = 0.570, -1.07 ms vs SHAM), indicating that active stimulation did not differ from SHAM.

Interaction effects were minimal across protocol groups: Visit and time interactions were found for conventional 1 Hz (F(1,370) = 5.99, p = 0.015) and TBS iTBS (F(1,379) = 4.08, p = 0.044), but no significant time x protocol interactions were observed for any protocol (conventional protocols: 1 Hz F(1,370) = 0.03, p = 0.44, 10 Hz F(1,375) = 0.177, p = 0.673; TBS protocols: iTBS F(1,379) = 0.12, p = 0.719, cTBS F(1,375) = 0.45, p = 0.502). No visit x protocol interactions were significant for any protocol group.

We found a main effect of time on completion time (CT); participants took longer to complete a sequence after rTMS. However, this difference was also found in the SHAM condition across all protocols, indicating it was not rTMS related. As expected, we found a main effect of hand, with the dominant hand faster to complete sequences than the non-dominant hand across all protocols.

ICC computations revealed poor reproducibility for most protocols, with only conventional 10 Hz showing moderate reproducibility for the ipsilateral hand (ICC = 0.52), while TBS protocols showed poor reproducibility for both hands (Table 1).

### 3.3 Correlation between measures and effect of baseline excitability on rTMS outcome

All correlations between rTMS effects on MEPs, EEG measures (P30, early LMFP), and SFTT (ipsilateral and contralateral hand) are given in Table 2. Across all protocol groups, correlations between measures were largely non-significant.

**Table 2.** Details of correlation results for each protocol, between EEG measures (P30 and early LMFP) and SFTT or MEP within each visit, each line represents a correlation between two measures.

Protocol	Correlation	Rho_V1	P_Value_V1	Rho_V2	P_Value_V2
1 Hz	P30x ipsilateral	0.0593	0.8403	-0.1648	0.5733
	P30x contralateral	-0.0066	0.9822	0.2967	0.303
	P30x MEP	-0.2598	0.3139	-0.0882	0.7363
	LMFPx ipsilateral	0.125	0.6326	0.1127	0.6666
	LMFPx contralateral	0.3284	0.1981	-0.0245	0.9256
	LMFPx MEP	-0.1203	0.6134	-0.1459	0.5395
10 Hz	p30x ipsilateral	-0.2647	0.3045	0.2029	0.451
	P30x contralateral	0.0662	0.8008	-0.3882	0.1373
	P30x MEP	-0.1275	0.6259	0.0753	0.7664
	LMFPx ipsilateral	-0.2921	0.2396	0.2265	0.399
	lmfpx contralateral	0.1703	0.4993	-0.5441	0.0293
	LMFPx MEP	-0.1022	0.6867	0.0237	0.9255
iTBS	P30x ipsilateral	0.4289	0.0858	-0.1838	0.48
	P30x contralateral	-0.348	0.171	-0.1887	0.4682
	P30x MEP	-0.1647	0.5421	-0.1529	0.5717
	LMFPx ipsilateral	0.0368	0.8886	-0.1275	0.6259
	LMFPx contralateral	-0.3554	0.1615	-0.2672	0.2999
	LMFPx MEP	-0.2412	0.3682	-0.1	0.7125
cTBS	P30x ipsilateral	-0.2529	0.3446	0.1588	0.5569
	P30x contralateral	-0.1607	0.5672	0.2643	0.3412
	P30x MEP	-0.2421	0.318	-0.0667	0.7863
	LMFPx ipsilateral	-0.1176	0.6643	0.1118	0.6803
	LMFPx contralateral	-0.0536	0.8496	0.2929	0.2895
	LMFPx MEP	-0.0228	0.9262	-0.2895	0.2293
SHAM	P30x ipsilateral	0.1022	0.6867	0.0506	0.8421
	P30x contralateral	-0.1187	0.639	0.3313	0.1793
	P30x MEP	0.4175	0.0753	-0.0737	0.7643
	LMFPx ipsilateral	0.2116	0.3994	0.0526	0.8357
	LMFPx contralateral	-0.1476	0.559	0.2384	0.3408
	LMFPx MEP	0.4088	0.0823	0.3544	0.1366

**Fig. 5.**
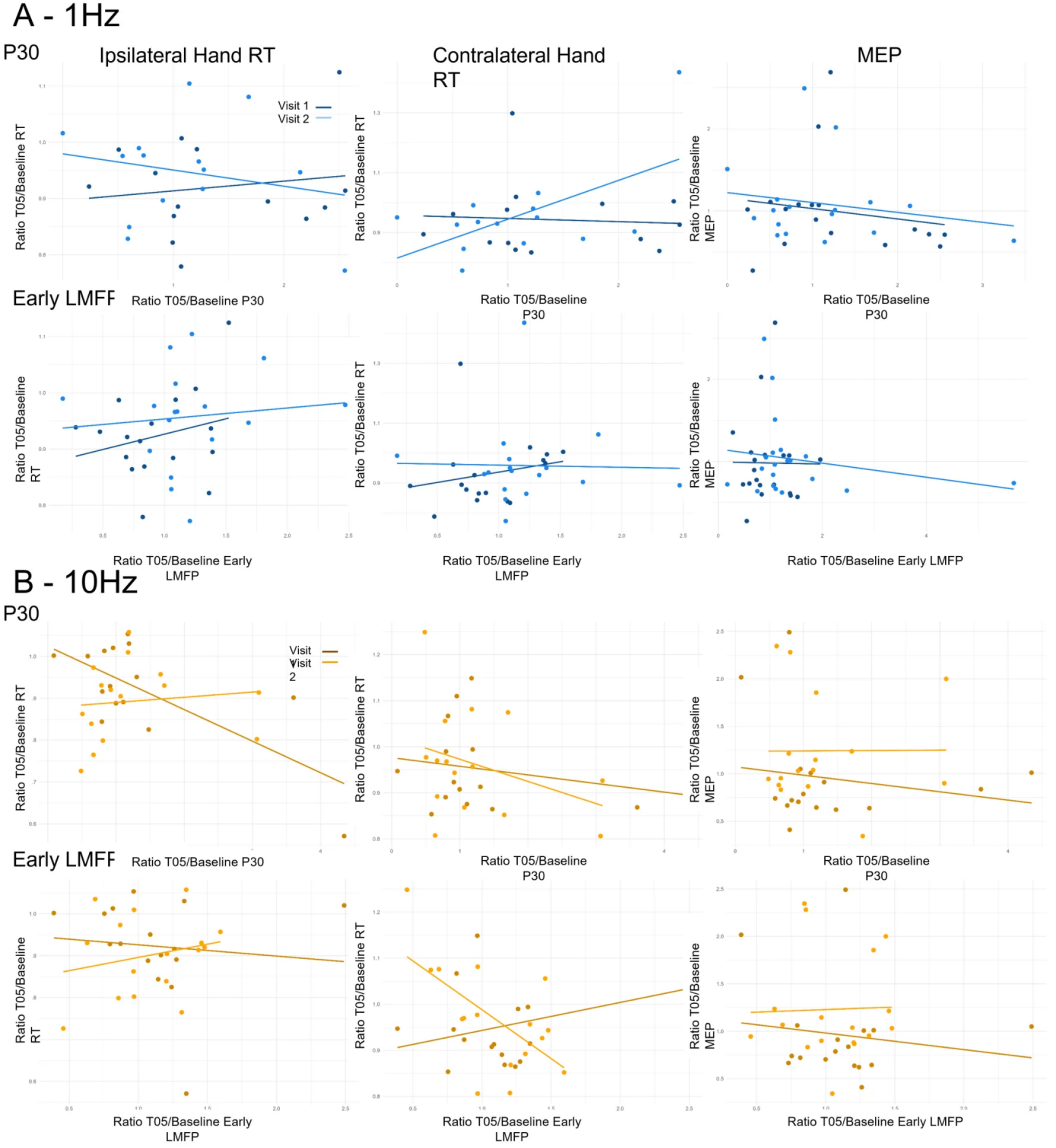
Scatter plot showing the convention rTMS protocols (A: 1 Hz, B: 10 Hz) correlation data between EEG measure (top row, P30, bottom row, early LMFP) and Ipsilateral hand CT (left panels), contralateral hand CT (middle panels) and MEP (right panels).

For conventional protocols and SHAM, there was a significant positive correlation between rTMS effects on contralateral RT and early LMFP in Visit 2 with 10 Hz stimulation (rho = -0.5441, p = 0.0293; Fig. 5), but this was not reproduced in Visit 1 and did not survive correction for multiple comparisons. No other significant correlations were found regardless of protocol (Fig. 5).

**Fig. 6.**
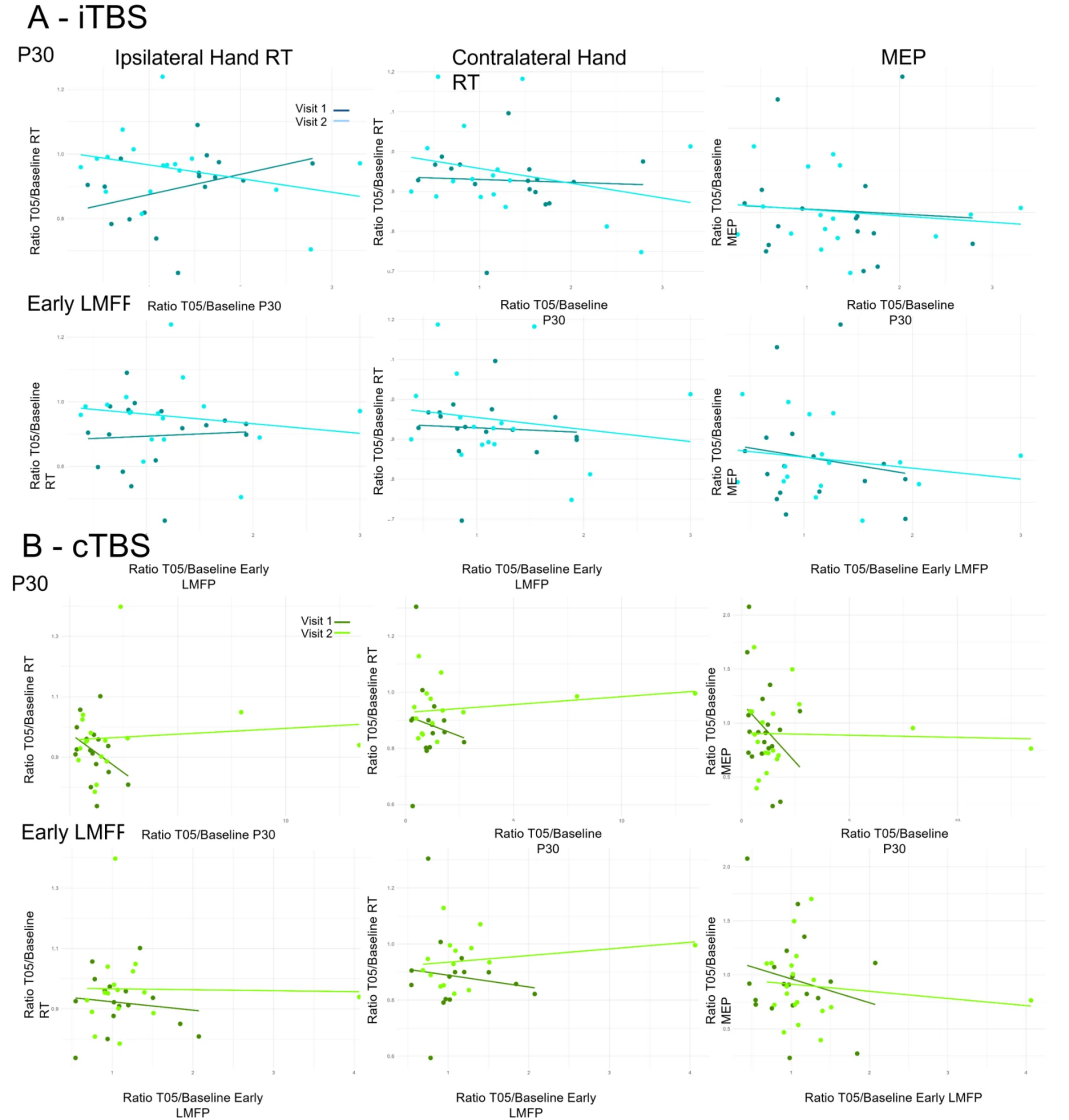
Scatter plot showing the theta-burst protocols (A: iTBS, B: cTBS) correlation data between EEG measure (top row, P30, bottom row, early LMFP) and ipsilateral hand CT (left panels), contralateral hand CT (middle panels), and MEP (right panels).

Similarly, TBS protocols showed no significant correlations between measures regardless of protocol type (Fig. 6).

Baseline excitability effects were minimal and non-reproducible across all protocol groups. For conventional protocols, some measures showed significant correlations with baseline excitability (such as 10 Hz where T25 P30 ratio showed negative correlation with baseline P30 in both visits, and MEP baseline amplitude correlated positively with T25 MEP ratio), but these did not survive multiple comparison correction and were not reproducible.

For TBS protocols, baseline P30 amplitudes correlated negatively with P30 changes for iTBS in both visits, but this did not survive multiple comparison correction and was not reliable (Fisher Z difference: F_Z = 0.2566, p = 0.79, p_bonferroni = 1) (Supplementary Table S1, Supplementary Fig. S6). Overall, we found no reproducible effect of baseline excitability on rTMS outcomes across any protocol group.

## 4 Discussion

Although initial studies reported consistent effects of rTMS protocols on cortical excitability, our findings challenge this characterization. We observed no significant group-level effects of any rTMS protocol (cTBS, iTBS, 10 Hz, or 1 Hz) on MEPs, TEPs, or task performance, along with considerable inter- and intra-subject variability across sessions, and with no consistent correlations between physiological and behavioral effects. These results challenge the common belief that rTMS influences brain function primarily through modulation of cortical excitability. Specifically, the assumption that rTMS protocols produce consistent “excitatory” or “inhibitory” effects that then drive behavioral or cognitive changes was not supported by our data.

Previous research in this area has significant methodological limitations. While meta-analyses have reported the expected inhibitory and excitatory effects of TBS on MEPs, the timing and magnitude of these effects have varied across studies, test–retest reliability is rarely assessed, and few studies include sham controls. Similarly, the literature on TEP modulation is inconsistent (Carrette et al., 2024; Moffa et al., 2022; Ozdemir et al., 2021), and systematic reviews of 10 Hz and 1 Hz rTMS physiological effects are scarce (Fitzgerald et al., 2006), with the last review highlighting substantial heterogeneity across studies. Our study addresses these gaps by systematically including sham controls and reliability assessments, finding that categorizing rTMS protocols simply as “excitatory” or “inhibitory” is likely oversimplified.

Our findings have important implications for studies using rTMS to modulate behavior. Based on models of interhemispheric inhibition, we hypothesized that inhibitory protocols (1 Hz and cTBS) would improve ipsilateral hand performance by reducing transcallosal inhibition from the stimulated to the non-stimulated motor cortex, thereby decreasing completion time. Conversely, we expected excitatory protocols (10 Hz and iTBS) to increase transcallosal inhibition and impair ipsilateral performance, thereby increasing completion time. This hypothesis was motivated by prior research demonstrating that inhibitory rTMS applied to M1 can enhance ipsilateral motor skill learning and performance through transcallosal disinhibition of the contralateral motor cortex (Kobayashi et al., 2004, 2009). However, contrary to these predictions, we found completion time increases following all protocols including sham, suggesting that the behavioral changes were not rTMS dependent. One explanation might be that the prior studies lacked sham conditions, and had substantially smaller sample sizes (Huang et al., 2005; Kobayashi et al., 2004). Furthermore, cognitive effects at the individual level were not associated with changes in cortical excitability, indicating that different mechanisms may underlie behavioral changes after rTMS.

A variety of recent TMS-fMRI and TMS-EEG studies have suggested that rTMS may induce changes in cerebral network connectivity with differential effects between TBS (Halko et al., 2014; Hermiller, 2024) and classical protocols (Grefkes et al., 2010). These connectivity changes may be responsible for the observed behavioral effects as some studies have shown links between changes in functional connectivity after parietal lobule stimulation and memory retrieval tasks (Battelli et al., 2017; Hermiller et al., 2019; Shafi et al., 2014; Warren et al., 2019).

Another potential mechanism of rTMS may be via modulation of cortical oscillatory activity (Hamidi, 2009); for example, 10 Hz rTMS applied to parieto-occipital alpha generators entrains cortical oscillations and can lead to changes in visual perception (Benwell et al., 2022; Thut et al., 2011). A study that applied iTBS using personalized theta and gamma frequencies for the pulse pattern reported greater effects on TEPs and behavior than conventional fixed-frequency iTBS (Chung et al., 2018), and changes in theta-gamma phase-amplitude coupling have been reported with iTBS (Zhang et al., 2023). Furthermore, rTMS effects based on the phase of brain oscillations at the time of stimulation have been shown in low-frequency rTMS, where triggering rTMS at the peak versus the trough of the ongoing Mu rhythm resulted in subsequent increase versus decrease in MEP amplitudes, respectively (Baur et al., 2020; Pillen et al., 2023). These studies highlight that rTMS does not operate in isolation but interacts with the ongoing oscillatory activity present at the time of stimulation.

Some studies have also suggested that rTMS modulates neurotransmitter levels (Ziemann et al., 2015), and can induce expression of neurotrophic factors such as Brain-Derived Neurotrophic factor (BDNF) (Cirillo et al., 2012; Gersner et al., 2011; Kim et al., 2016; Pan et al., 2023; Zhao et al., 2019); for example, following a 7-day iTBS procedure, BDNF levels were significantly higher than sham-iTBS (Pan et al., 2023). However, some studies show little to no rTMS effects on BDNF (Gedge et al., 2012). Studies evaluating the evolution of BDNF following each session might help uncover the plasticity mechanisms involved in rTMS (Ørbo et al., 2023; Sanna et al., 2022). Other studies have suggested that rTMS may have non-neuronal effects on glia and inflammatory cascade (Cullen & Young, 2016; Ferreira et al., 2023). A number of other potential mechanisms have been reported in animal models, but need further assessment and validation.

A key consideration is that clinical rTMS protocols typically involve multiple sessions. Recent clinical trials showing positive outcomes for depression have not clarified whether cortical excitability changes are involved (Cole et al., 2022; Morris et al., 2020). While single rTMS sessions may not reliably alter excitability, multiple sessions might produce cumulative effects. However, a study examining five daily iTBS sessions found no significant changes in cortical excitability compared with sham stimulation (Cole et al., 2022), suggesting that the relationship between excitability and clinical efficacy remains unclear (Perellón-Alfonso et al., 2018).

In the context of the widespread replication crisis across neuroimaging (Button et al., 2013; Eklund et al., 2016; Marek et al., 2022) and as the TMS field trends toward personalization of protocols, our results emphasize the need for systematic studies that establish the reliability of rTMS effects. Studies exploring individualized targeting (e.g., based on connectome resting-state fMRI, individualized fMRI, or DTI) or other innovations in TMS need to include both sham stimulation and reproducibility measures to establish reliable TMS-specific effects.

### 4.1 Limitations

Several limitations should be recognized. First, focusing solely on M1 may not capture the effects of rTMS on other areas involved in more complex cognitive or emotional processing. Although our study tested the most utilized rTMS protocols, many studies reporting “inhibitory” or “excitatory” effects, as well as most clinical trials, are done with stimulation of the DLPFC. Second, the small number of trials at the T25 time point limited our analysis, though the available data showed similar variability patterns. Third, our TMS/EEG results focused on local excitability on the scalp in the temporal domain to characterize the local changes induced by rTMS. However, some other studies have reported modulatory effects in the time–frequency domain or in the source domain (Chung et al., 2017, 2018); the reliability and reproducibility of these effects remain to be tested. Some studies have found effects of rTMS on later components of the TEP, such as the N100–P200 complex (Roos et al., 2021; Ye et al., 2022). However, the lack of realistic sham control in most of these studies, and the presence of prominent sensory-evoked potentials at those time points, complicates interpretation of those results. Fourth, despite controlling many variables, individual differences in anatomy, coil positioning, and effective stimulation likely contributed to response variability. Additionally, our study design prioritized maintaining consistent targeting of the FDI motor hotspot to ensure reliable targeting of the FDI hotspot for alignment with our behavioral task and measurement of MEPs. Although this design choice was necessary for our multi-modal outcome approach, it limited the degrees of freedom in terms of maximizing TEP signal-to-noise ratios, which might have contributed to the variability seen in TEP analyses (Casarotto et al., 2022). Another limitation is that the SFTT task might have been too easy to produce a significant detectable effect after a single rTMS session.

## 5 Conclusion

In this study, we systematically evaluated the effects and test–retest reliability of the four most commonly used rTMS protocols (10 Hz rTMS, 1 Hz rTMS, iTBS, cTBS) relative to sham rTMS on corticospinal excitability, cortical excitability, and behavioral task performance. We found no group-level and almost no individually reliable effects of any rTMS protocol, relative to sham stimulation, and we found no correlations between cortical excitability changes and behavioral effects. Our study challenges the traditional view of rTMS as inducing “excitatory” or “inhibitory” changes in brain excitability, and suggests the need for a fundamental unbiased re-evaluation of potential rTMS mechanisms. To further the understanding of rTMS effects and make sense of the literature, future research should include reproducibility measures and a realistic-sham control condition.

## Supplementary Material

Supplementary Material

## Data Availability

Raw de-identified data files are already uploaded to National Institute of Mental Health archive in accordance with the rules of the local ethics committee and can be accessed in the following link: https://nda.nih.gov/edit_collection.html?id=2944
