# Supplementary material for: Assessing the efficacy and reproducibility of four common repetitive transcranial magnetic stimulation protocols: A sham-controled study
Source: Imaging Neurosci (Camb). 2026 Jul 16;4:IMAG.a.1231. doi: 10.1162/IMAG.a.1231 (PMC13377501; doi:10.1162/IMAG.a.1231)
Supplement: Supplementary Material [file IMAG.a.1231_supp.pdf]

Omnibus Testing

LMFP

The omnibus tests revealed no main effects of Protocol ($F(4,497) = 0.294$, $p = .882$), Visit ($F(1,490) = 3.028$, $p = .082$), or Time ($F(2,492) = 0.129$, $p = .879$). However, a significant Protocol × Visit interaction emerged ($F(4,449) < .001$). The Protocol × Time ($F(8,491) = 0.317$, $p = .960$), Visit × Time ($F(2,490) = 0.352$, $p = .703$), and three-way Protocol × Visit × Time ($F(8,490) = 0.140$, $p = .997$) interactions were not significant.

Post-hoc examination of the Protocol × Visit interaction showed that iTBS exhibited a significant increase from V1 to V2 compared to 10Hz ($\beta = 63.56$, SE = 30.42, 95% CI [3.94, 123.19], $t(490.3) = 2.089$, $p = .037$). The cTBS protocol showed a trend toward decreased amplitude at V2 relative to 10Hz ($\beta = -52.01$, SE = 30.51, 95% CI [-111.82, 7.80], $t(490.3) = -1.704$, $p = .089$). No other pairwise Protocol × Visit comparisons reached significance.

P30 Amplitude

Omnibus tests showed no main effects of Protocol ($F(4,487) = 0.944$, $p = .438$), Visit ($F(1,479) = 0.307$, $p = .580$), or Time ($F(2,481) = 0.220$, $p = .803$). The Protocol × Visit interaction was highly significant ($F(4,479) < .001$), while Protocol × Time ($F(8,479) = 0.294$, $p = .968$), Visit × Time ($F(2,478) = 0.268$, $p = .765$), and the three-way interaction ($F(8,478) = 0.205$, $p = .990$) were not significant.

Within the Protocol × Visit interaction, iTBS showed a robust increase from V1 to V2 compared to 10Hz ($\beta = 2.115$, SE = 0.625, 95% CI [0.889, 3.340], $t(478.4) = 3.383$, $p < .001$). No other protocol comparisons showed significant differential changes across visits (all $p > .390$).

SFTT

Omnibus tests revealed no significant main effects: Protocol ($F(4,326) = 0.113$, $p = .978$), Visit ($F(1,316) = 3.716$, $p = .055$), or Time ($F(1,312) = 1.475$, $p = .225$). Similarly, all interaction terms were non-significant: Protocol × Visit ($F(4,314) = 1.166$, $p = .326$), Protocol × Time ($F(4,312) = 1.362$, $p = .247$), Visit × Time ($F(1,312) = 0.186$, $p = .667$), and the three-way interaction ($F(4,312) = 0.830$, $p = .507$).

The SHAM protocol showed a marginal increase in reaction time at T05 relative to 10Hz ($\beta = 0.075$, SE = 0.039, 95% CI [-0.001, 0.152], $t(312.0) = 1.928$, $p = .055$), but this did not survive correction for multiple comparisons. No other effects approached significance.

MEP

Omnibus tests showed no significant main effects of Protocol ($F(4,347) = 0.543$, $p = .704$), Visit ($F(1,338) = 1.070$, $p = .302$), or Time ($F(1,338) = 0.676$, $p = .411$). All interactions were likewise non-significant: Protocol × Visit ($F(4,338) = 1.369$, $p = .244$), Protocol × Time ($F(4,338) = 0.067$, p

= .992), Visit × Time (F(1,338) = 0.224, p = .636), and the three-way interaction (F(4,338) = 0.097, p = .983).

Individual parameter estimates revealed no significant differences between protocols at either visit or time point (all p > .436), indicating that MEP amplitudes remained stable across experimental manipulations.

Supplemental Hjorth-C3 Montage Analysis:

P30 Amplitudes for all trials

The general mixed-effects model analysis of the P30 peak amplitude in the Hjorth-C3 montage (Figure 1 supplementary) revealed no significant main effects of Visit (F(1, 354) = 1.445, p = .230), Time (Baseline vs T5; F(1, 351) = 0.469, p = .494), or Protocol (F(4, 360) = 2.330, p = .056). Additionally, no significant interactions were observed: Visit × Time (F(1, 351) = 0.382, p = .537), Visit × Protocol (F(4, 354) = 2.320, p = .057), Time × Protocol (F(4, 351) = 0.166, p = .955), or the three-way Visit × Time × Protocol interaction (F(4, 351) = 0.281, p = .890). .

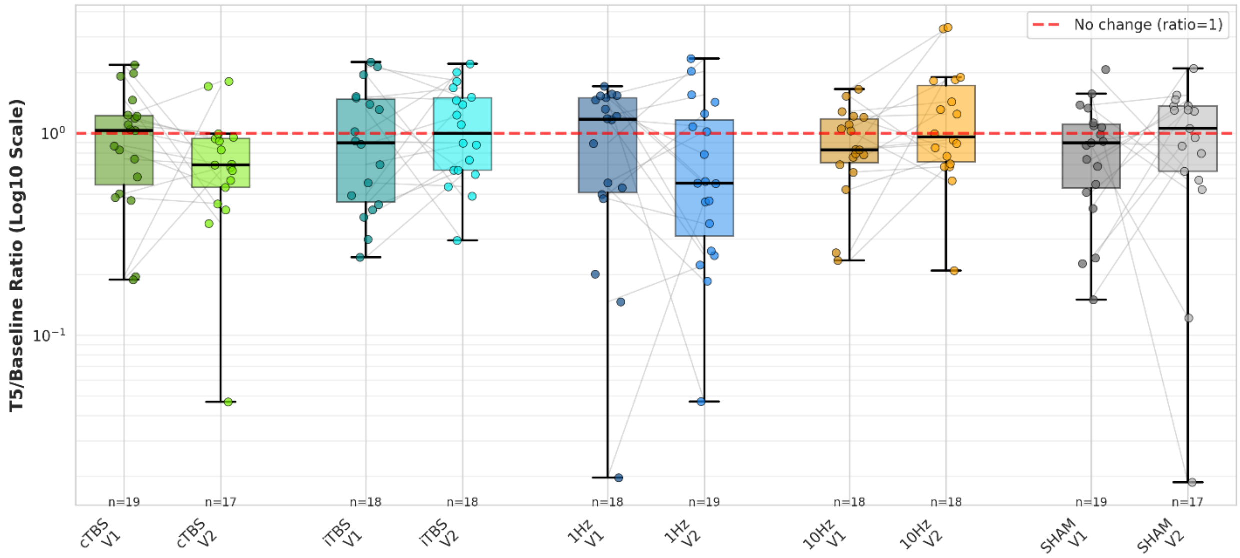

Figure 1 supplementary. Effect of rTMS on C3-Hjorth montage. Plot of the log10 of the Ratio between pre and post rTMS for both visit (V1 - darker, V2 - lighter shade) and each protocol (1Hz, blue - 10Hz, orange - SHAM, gray).

Hjorth-C3 RMS for all Trials

The general mixed-effects model analysis of RMS (AllTrials) revealed no significant main effects of Protocol (F(4, 365) = 0.895, p = .467), Visit (F(1, 358) = 2.043, p = .154), or Condition (F(1, 355) = 0.314, p = .576). Additionally, no significant interactions were observed: Protocol × Visit (F(4, 361) = 2.046, p = .087), Protocol × Condition (F(4, 355) = 1.124, p = .345), Visit × Condition (F(1, 355) =

1.066, p = .302), or the three-way Protocol × Visit × Condition interaction (F(4, 355) = 1.259, p = .286). The Protocol × Visit interaction approached but did not reach significance (p = .087).

<u>Hjorth-C3 Window Size 40 trials analysis</u>

P30

The general mixed-effects model analysis of the P30 amplitude (Figure 2 supplementary) for the Hjorth-C3 montage revealed a significant main effect of Protocol (F(4, 548) = 3.660, p = .006) and a significant Visit x Protocol interaction (F(4, 542) = 3.795, p = .005). The main effect of Condition (Baseline vs TF, Baseline vs TL, TF vs TL) was not significant (F(1,549)=0.454, p=0.501). The main effect of Visit was marginal (F(1, 542) = 3.609, p = .058), while the main effect of Window (F(2, 539) = 0.640, p = .528), the Visit x Window interaction (F(2, 539) = 0.179, p = .836), the Window x Protocol interaction (F(8, 539) = 0.169, p = .995), and the three-way interaction (F(8, 539) = 0.327, p = .956) were not significant. Post-hoc comparisons of the significant main effect of Protocol indicated that 1Hz yielded significantly lower Peak Amplitude compared to both iTBS (M_Diff = -0.838, p = .020) and SHAM (M_ Diff = -0.870, p = .011).

RMS

The general mixed-effects model analysis of RMS (40 trials) revealed no significant main effect of Condition (Baseline vs TF, Baseline vs TL, TF vs TL; F(2, 541) = 0.278, p = .757) or Protocol (F(4, 553) = 1.655, p = .159), but a significant main effect of Visit (F(1, 545) = 4.509, p = .034). A significant Protocol × Visit interaction was observed (F(4, 548) = 3.687, p = .006), but no significant Condition × Protocol (F(8, 541) = 0.773, p = .627), Condition × Visit (F(2, 541) = 0.553, p = .575), or three-way Condition × Protocol × Visit interaction (F(8, 541) = 0.764, p = .634) was found. Post-hoc comparisons of the significant Protocol × Visit interaction indicated that 1Hz showed a significantly larger decrease from V1 to V2 compared to 10Hz (M_Diff = -146.58, p = .002), as did cTBS compared to 10Hz (M_Diff = -145.17, p = .002).

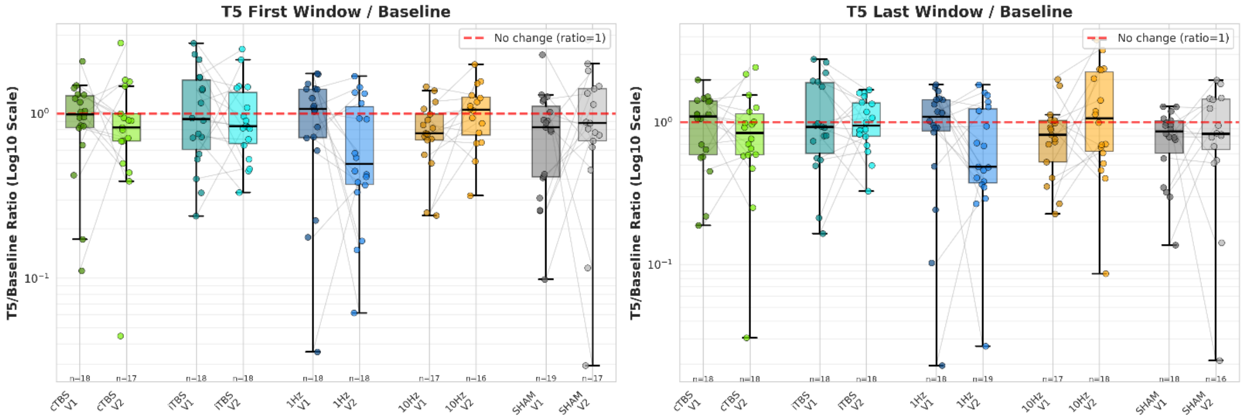

Figure 2 supplementary. Effect of rTMS on C3-Hjorth montage P30 amplitude for: left, ratio of last 40 trials of Baseline vs first 40 trials of T5; right, ratio of last 40 trials of Baseline vs last 40 of T5.

Supplemental N15-P30

The omnibus analyzing the N15-P30 amplitude across all protocols (N = 367 observations, 27 subjects) showed no significant main effects for Protocol (F(3,276.07) = 0.6408, p = .5893), Time (F(1,273.22) = 1.3286, p = .2501), or Visit (F(1,276.99) = 0.1426, p = .7060). Furthermore, none of the interaction terms reached statistical significance: Protocol x Time (F(3,273.22) = 0.5447, p = .6521), Protocol x Visit (F(3,275.25) = 1.6297, p = .1827), Time x Visit (F(1,273.22) = 0.2089, p = .6480), or the three-way Protocol x Time x Visit interaction (F(3,273.23) = 0.1071, p = .9559).

Analysis of 40-Trial Windows

LMFP Analysis: 40 trials

Omnibus test for the LMFP in windows of 40 trials (Figure 3 supplemental) revealed that the main effects of Protocol (F(4, 546) = 0.998, p = .408), Visit (F(1, 542) = 1.425, p = .233), and Condition (Baseline vs TF, Baseline vs TL, TF vs TL): F(1, 539) = 2.045, p = .153) Interactions were not statistically significant.

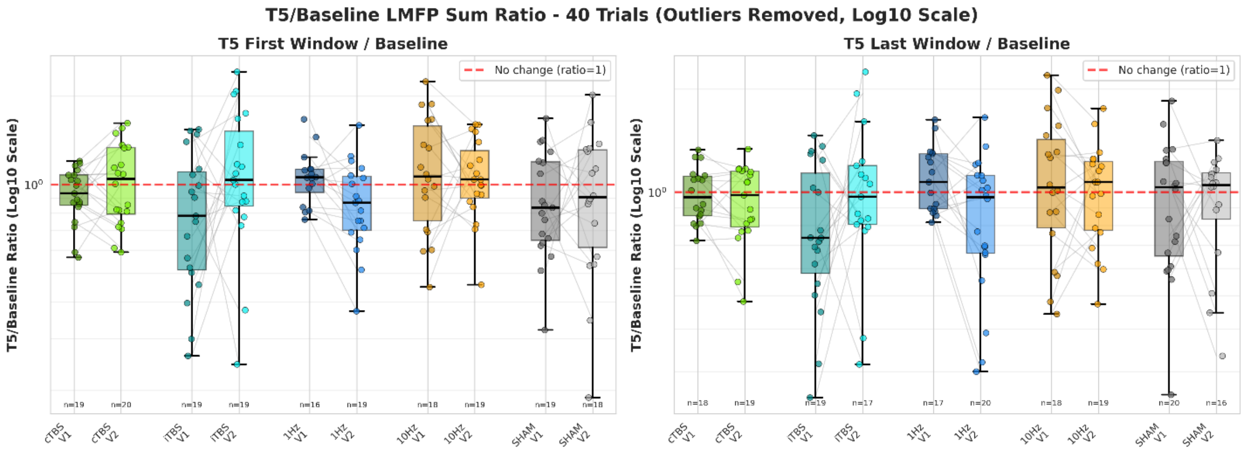

Figure 3 supplementary. Effect of rTMS on LMFP between left: ratio of last 40 trials of baseline vs first 40 trials of T5 and right: ratio of last 40 trials of Baseline vs last 40 of T5.

P30 Analysis: 40 trials

For P30 peak amplitude divided in 40 trials (Figure 4 supplemental) we see no significant main effects (Protocol: F(4, 546) = 1.735, p = .141; Visit: F(1, 541) = 2.485, p = .116; Condition (Baseline vs TF, Baseline vs TL, TF vs TL)): F(1, 539) = 0.517, p = .473) or interactions (Protocol x Visit: F(4, 541) = 1.040, p = .386; all others p > .27)

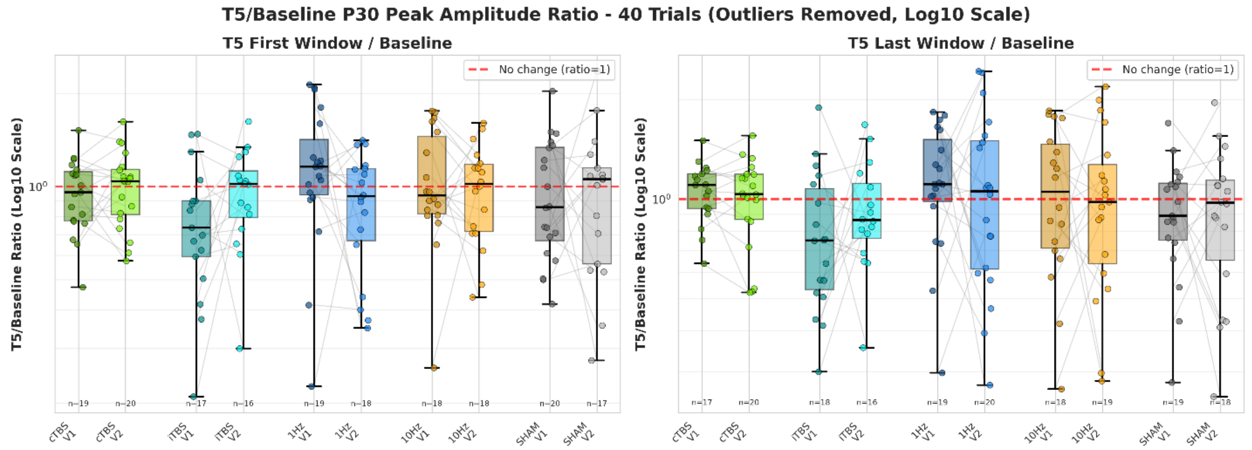

Figure 4 supplementary. Effect of rTMS on P30 between left: ratio of last 40 trials of baseline vs first 40 trials of T5 and ratio of last 40 trials of Baseline vs last 40 of T5.

<u>Time Series Analysis: 40 trials</u>

For the time series comparison (Figure 5 supplemental), we performed the same cluster-based permutation analysis presented in the main manuscript. We repeated the analysis using a 40-trial window for each condition and outcome measure (LMFP, GMFP, C3-Hjorth). Similarly to the full dataset, the 40-trial window analysis showed no significant time points regardless of protocol (all $p > .05$, cluster-corrected). This consistency suggests that the lack of differential TEP modulation across visits was not attributable to variability in time of the window during the recordings (first 40 trials or last 40 trials) across protocols or participants.

For illustration purposes, we present below a representative comparison for the iTBS protocol between V1 and V2 (Figure 5 supplementary). The complete time series analyses for all protocols and visits, including both full and 40-trial window datasets, may be found in the online data repository.

<u>N15-P30 Analysis: 40 trials</u>

For N15-P30 peak-to-peak amplitude, we observed no significant main effects (Protocol: $F(4, 750) = 1.944$, $p = .102$; Visit: $F(1, 743) = 2.061$, $p = .152$; Condition: $F(1, 735) = 2.101$, $p = .148$) or interactions (Protocol × Visit: $F(4, 743) = 1.804$, $p = .126$; Protocol × Condition: $F(4, 735) = 2.024$, $p = .090$; Visit × Condition: $F(1, 736) = 2.071$, $p = .150$; Protocol × Visit × Condition: $F(4, 735) = 1.914$, $p = .106$).

LMFP

V1

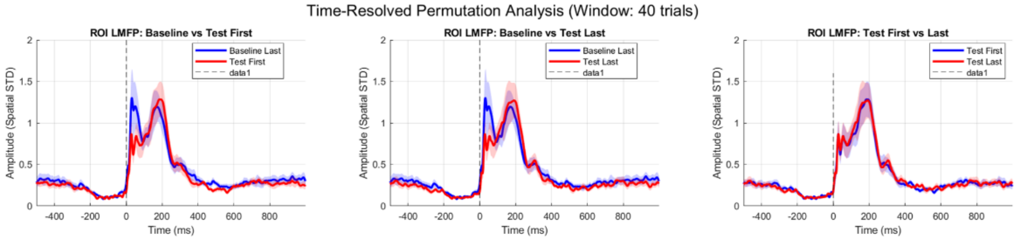

V2

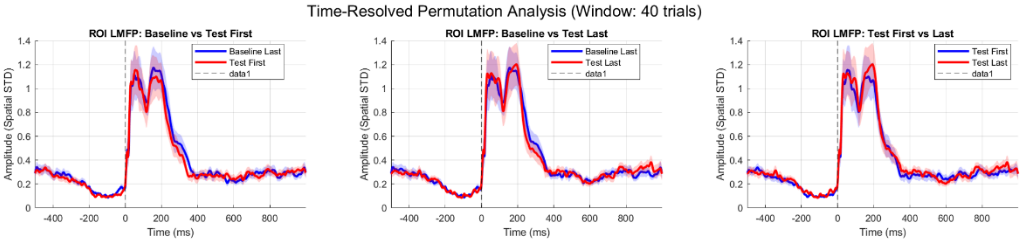

GMFP

V1

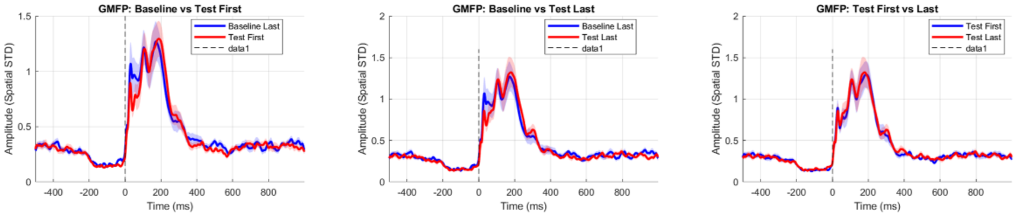

V2

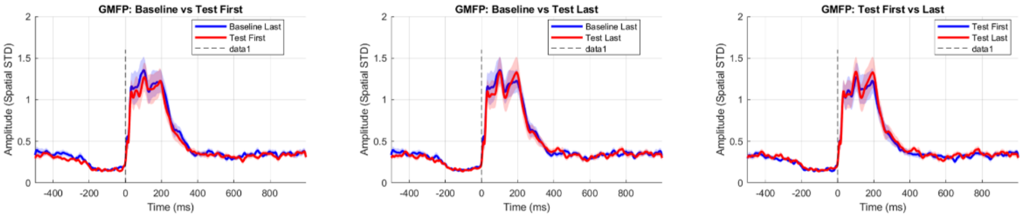

C3-Hjorth

V1

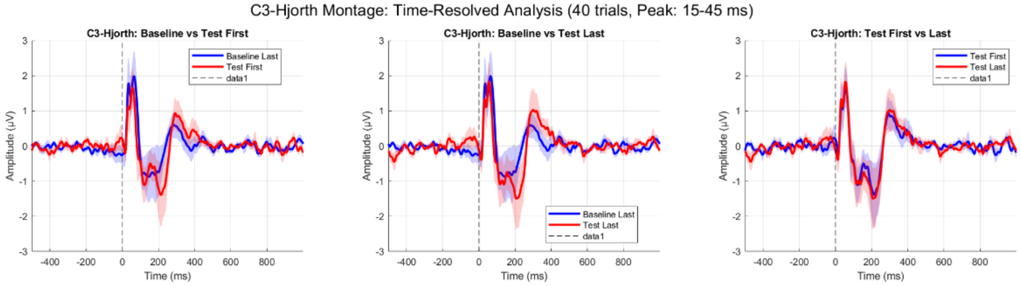

V2

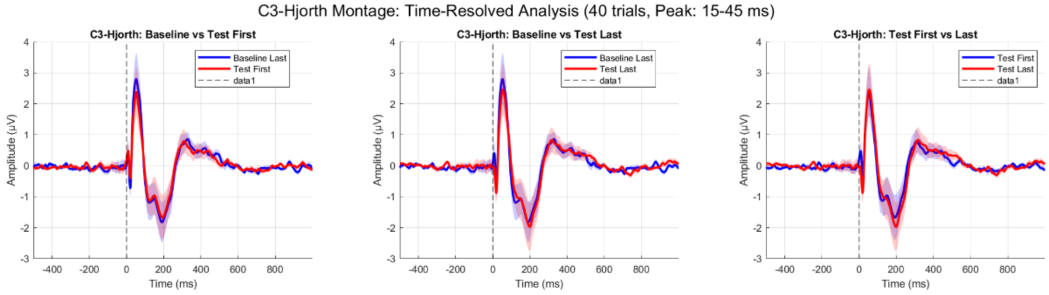

Figure 5 supplemental Time series of LMFP, GMFP and C3-Hjorth showing in blue average TEP for the last 40 trials of Baseline and in red Left: First 40 trials of T5, Middle Last 40 trials of T5 and Right TEP overlay of First and Last 40 trials of T5.

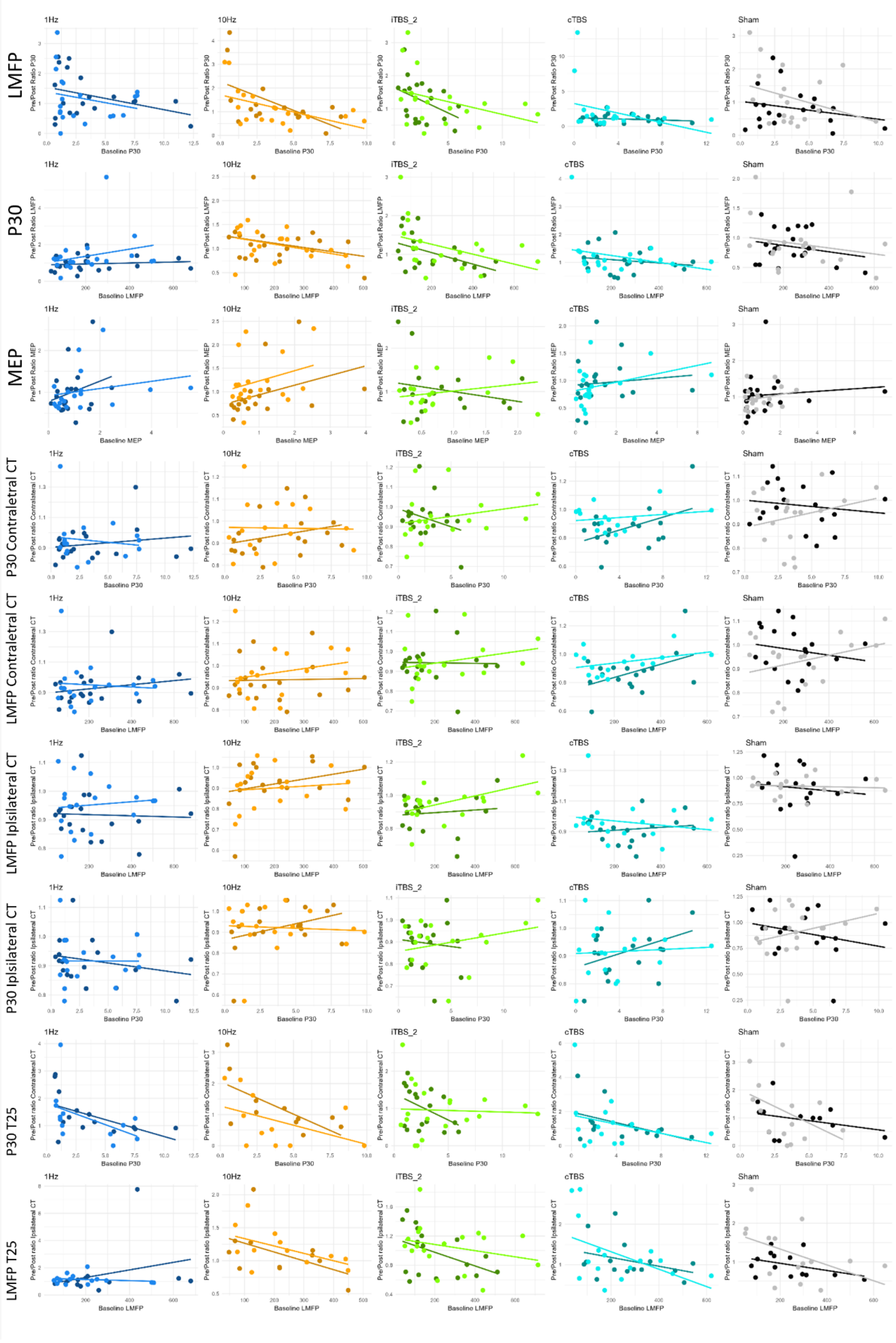

LMFP
P30
MEP
P30 Contralateral CT
LMFP Contraletral CT
LMFP Iplsilateral CT
P30 Iplsilateral CT
P30 T25
LMFP T25
1Hz
10Hz
iTBS_2
cTBS
Sham
Pre/Post Ratio P30
Pre/Post Ratio LMFP
Pre/Post Ratio MEP
Pre/Post ratio Contralateral CT
Pre/Post ratio Ipsilateral CT
Baseline P30
Baseline LMFP
Baseline MEP

Figure 6. supplementary Scatter plot showing the correlation between Baseline measure excitability (P30,LMFP) and effect of rTMS (Ratio of the different outcomes measures: Row 1: P30, Row 2: LMFP, Row3: MEP, Rows 4 and 5: Contralateral CT, Rows 6 and 7: Ipsilateral CT) for each protocol (Blue 1Hz, Orange 10Hz, Green iTBS, Teal cTBS, Black Sham)

Baseline correlations	Protocol	Spearman_Rho_V1	P_Value_V1	Bonferroni_P_V1	Spearman_Rho_V2	P_Value_V2	Bonferroni_P_V2	Fisher_Z_Diff	Fisher_P_Value	Bonferroni_Fisher_P
LMFP	1Hz	-0.19	0.4329	1	-0.3	0.1976	0.9879	0.3197	0.7492	1
	10Hz	-0.51	0.0303	0.1514	-0.56	0.0121	0.0603	0.2015	0.8403	1
	iTBS	-0.41	0.078	0.3901	-0.47	0.044	0.2199	0.1848	0.8534	1
	cTBS	-0.11	0.6587	1	-0.22	0.349	1	0.3473	0.7284	1
	SHAM	-0.14	0.5565	1	-0.36	0.123	0.6149	0.6763	0.4988	1
P30	1Hz	0.19	0.417	1	0.27	0.2317	1	-0.2717	0.7859	1
	10Hz	-0.24	0.3289	1	-0.37	0.115	0.5751	0.4279	0.6688	1
	iTBS	-0.5	0.0293	0.1463	-0.56	0.0117	0.0586	0.2566	0.7975	1
	cTBS	-0.12	0.6001	1	-0.28	0.2376	1	0.4625	0.6437	1
	SHAM	-0.09	0.7005	1	-0.26	0.2623	1	0.5175	0.6048	1
MEP	1Hz	0.24	0.2862	1	0.43	0.0574	0.2871	-0.62	0.5353	1
	10Hz	0.58	0.0115	0.0573	0.15	0.5479	1	1.3523	0.1763	0.8815
	iTBS	-0.14	0.5863	1	0.09	0.717	1	-0.5997	0.5487	1
	cTBS	0.31	0.2011	1	0.39	0.101	0.5048	-0.2598	0.795	1
	SHAM	0.27	0.2457	1	0.23	0.3515	1	0.1384	0.89	1
P30 Contraletral CT	1Hz	0.17	0.4861	1	-0.19	0.4623	1	0.8951	0.3708	1
	10Hz	0.36	0.1421	0.7103	0.17	0.5276	1	0.5221	0.6016	1
	iTBS	-0.19	0.458	1	0.4	0.1145	0.5727	-1.6117	0.107	0.5351
	cTBS	0.09	0.7292	1	-0.08	0.7783	1	0.4104	0.6815	1
	SHAM	-0.15	0.5534	1	0.15	0.5424	1	-0.8374	0.4024	1
LMFP Contraletral CT	1Hz	0.28	0.2246	1	0.15	0.5645	1	0.3855	0.6999	1
	10Hz	0.01	0.9838	1	0.27	0.3163	1	-0.6862	0.4926	1
	iTBS	0.07	0.7851	1	0.26	0.3139	1	-0.5203	0.6029	1
	cTBS	0.09	0.7222	1	0.18	0.4991	1	-0.2229	0.8236	1

	SHAM	-0.22	0.3762	1	0.18	0.4682	1	-1.1238	0.2611	1
LMFP Iplsilateral CT	1Hz	-0.12	0.6318	1	0.07	0.7851	1	-0.4957	0.6201	1
	10Hz	0.27	0.276	1	0.21	0.4443	1	0.1772	0.8593	1
	iTBS	0.05	0.8518	1	0.38	0.138	0.6902	-0.9132	0.3611	1
	cTBS	0.03	0.9182	1	-0.13	0.6259	1	0.3955	0.6925	1
	SHAM	-0.18	0.4682	1	-0.16	0.5369	1	-0.0757	0.9397	1
P30 Iplsilateral CT	1Hz	-0.16	0.5315	1	0.02	0.9384	1	-0.4732	0.6361	1
	10Hz	0.35	0.1573	0.7865	-0.29	0.2431	1	1.8115	0.0701	0.3503
	iTBS	-0.17	0.5164	1	0.18	0.486	1	-0.937	0.3487	1
	cTBS	0.24	0.3582	1	0.06	0.8153	1	0.479	0.632	1
	SHAM	-0.26	0.3033	1	0.37	0.1302	0.6508	-1.7851	0.0742	0.3712
P30 T25	1Hz	-0.57	0.032	0.1599	-0.65	0.0153	0.0767	0.2415	0.8092	1
	10Hz	-0.71	0.0146	0.0728	-0.62	0.0404	0.2021	-0.2189	0.8267	1
	iTBS	-0.45	0.0709	0.3547	0.06	0.7972	1	-1.4449	0.1485	0.7424
	cTBS	-0.49	0.0664	0.3321	-0.24	0.3831	1	-0.632	0.5274	1
	SHAM	-0.38	0.1944	0.9721	-0.46	0.0985	0.4923	0.1931	0.8469	1
LMFP T25	1Hz	0.32	0.2427	1	-0.15	0.5936	1	1.136	0.256	1
	10Hz	-0.42	0.2006	1	-0.56	0.0586	0.2929	0.349	0.7271	1
	iTBS	-0.42	0.0898	0.4492	-0.17	0.4993	1	-0.7425	0.4578	1
	cTBS	-0.33	0.2318	1	-0.4	0.1248	0.6238	0.2019	0.84	1
	SHAM	-0.35	0.2269	1	-0.44	0.1045	0.5225	0.2512	0.8017	1

Table 1. supplementary: Details of correlations results for each protocol and combinations of baseline excitability (P30, LMFP) and outcome measure. Including reproducibility of correlation (Fischer Z).
